# Modeling and compensation of hysteresis in piezoelectric actuators

**DOI:** 10.1016/j.heliyon.2020.e03999

**Published:** 2020-05-30

**Authors:** Zhiliang Yu, Yue Wu, Zhiyi Fang, Hailin Sun

**Affiliations:** a Aerospace System Engineering Shanghai, Shanghai 201109, China; b School of Astronautics, Harbin Institute of Technology, Harbin 150001, China

**Keywords:** Electrical engineering, Physics, Piezoelectric actuator, Precision positioning, Hysteresis, Prandtl-Ishlinskii

## Abstract

Piezoelectric actuator has the advantages of high rigidity, wide bandwidth, fast response and high resolution. Therefore, they are widely used in many micro and nano positioning applications. However, the hysteresis characteristic in the piezoelectric actuator (PEA) seriously affects its positioning accuracy and even causes instability. In this paper, a modified Prandtl-Ishlinskii (MPI) model, which can describe the rate asymmetric hysteresis of piezoelectric actuator, is studied. The hysteresis compensation is realized by using the rate dependent Prandtl-Iishlinskii model based on the improved Prandtl-Iishlinskii hysteresis model and the hysteresis characteristics of the driver measured in the laboratory under the frequency input of up to 100 Hz. In order to further reduce the error of feedforward compensation, a sliding mode controller is designed. The stability of the control system is proved by Lyapunov theory. The experimental results show that the linear error of the system is reduced from 10% to less than 1%, and the tracking error can also be reduced by 90%.

## 1 Introduction

Piezoelectric actuator has the advantages of large output force, wide frequency band and fast frequency response. Piezoelectric actuators play an important role in micro nano applications [1]. However, as a kind of polar material, piezoelectric actuator often shows the nonlinear hysteresis between input current and output displacement. At high input rate, the hysteresis nonlinearity is often more significant, which will not only directly affect the accuracy of high-precision positioning, but also lead to response oscillation and error, resulting in poor tracking performance of the closed-loop system [2]. Generally, it is necessary to identify the hysteresis model and the compensation controller based on the inverse hysteresis model to realize the high-precision positioning control of pea, and the establishment of the hysteresis model and parameter identification play a fundamental and important role in the establishment of the accurate hysteresis model.

In order to solve this problem, many hysteresis models describing the hysteresis nonlinearity of piezoelectric actuator are proposed in the literature, such as Preisach model [3], Krasnosel'skii-Pokrovskii model [4], Prandtl-Ishlinskii model and differential equation based hysteresis model, such as backlash model, Duhem model, Bouc-Wen model [5]. However, few studies show that these hysteretic models can describe symmetric and rate independent hysteretic, but in practical applications, hysteretic models show asymmetry and dependence.

An improved sliding mode motion tracking control method for piezoelectric actuator is proposed. A control method for parameter uncertainty, nonlinearity (including hysteresis effect) and other un-modelled disturbances is proposed, and the asymmetric hysteresis characteristics of generalized play operator are described by using the improved PI model (MPI). This MPI has been used in model-based compensation schemes. A feedforward control method based on MPI model is proposed. In order to further reduce the influence of compensation error, parameter uncertainty and external interference, based on the identification model of piezoelectric actuator, the proposed sliding mode control method is established, and the proposed control method is analyzed. The stability of the proposed control method is proved theoretically, and the performance of the proposed scheme is verified by the experimental results. The scheme can compensate the hysteresis effect.

## 2 Hysteresis mathematical model

In this section, we mainly discuss how to use generalized hysteresis operator to describe MPI model and how to design feedforward controller to compensate the actuator's hyste-resis nonlinearity.

### 2.1 Modified PI model

The classical PI model can't describe the asymmetric hysteresis. In order to solve this problem, a generalized nonlinear player is used. The generalized play operator $\varpi^{\prime }\left(t\right)=P_{r}^{\prime }\left[u\right]\left(t\right)$ is defined as follows:

(1) $$\left\{ \begin{array}{l} \varpi^{\prime }\left(0\right)=P^{\prime }\left[u\right]\left(0\right)=f^{\prime }\left(u\left(0\right),0\right)\\ \varpi^{\prime }\left(t\right)=P^{\prime }\left[u\right]\left(t\right)=f^{\prime }\left(u\left(t\right),\varpi^{\prime }\left(t_{k}\right)\right);\\ t_{k}\leq t<t_{k+1},0\leq k\leq N\\ f^{\prime }\left(u,\varpi^{\prime }\right)=\max\left\{\begin{array}{l} a\;\exp\left(\tau u-br\right)+c,\\ \min\left(a^{\prime }\;\exp\left(\tau^{\prime }u+b^{\prime }r\right)+c^{\prime },\varpi^{\prime }\right) \end{array}\right\} \end{array}\right.$$

(2) $$\left\{ \begin{array}{l} a\neq a^{\prime }\neq 0\\ b\neq b^{\prime }\neq 0\\ c\neq c^{\prime }\neq 0 \end{array}\right.$$

where $\tau ,\tau^{\prime },a,a^{\prime },b,b^{\prime },c,c^{\prime }$are unknown constants that need to be determined.

rrepresents the thresholds on both sides of the generalized play operator. Two different threshold and envelope functions can describe the asymmetric hysteresis loop. These constants corresponding to the increase and decrease of input are related to the envelope function and threshold value, and their relations are as follows:

(3) $$\left\{ \begin{array}{l} \zeta_{1}\left(r\right)=\alpha rfor\dot{u}\left(t\right)>0\\ \zeta_{2}\left(r\right)=\beta \left(-r\right)for\dot{u}\left(t\right)<0 \end{array}\right.$$

where αandβ are $\phi \left(\dot{u}\right)$ and $\phi \left(\dot{u}\right)$. The generalized play hysteresis operator is determined with the given threshold r.

Based on the generalized play operator, the MPI model can be given by

(4) $$y\left(t\right)=h\left(u\right)+\int_{0}^{+\infty }p\left(r\right)P^{\prime }\left[u\right]\left(t\right)dr$$

where $h\left(u\right)=a_{1}u^{3}+a_{2}u^{2}+a_{3}u$, and $a_{1},a_{2},a_{3}$ are polynomial coefficients.

(5) $$\left[\begin{array}{l} y_{1}\\ y_{2}\\ \vdots \\ y_{m} \end{array}\right]=a_{1}\left[\begin{array}{l} u_{1}^{3}\\ u_{2}^{3}\\ \vdots \\ u_{m}^{3} \end{array}\right]+a_{2}\left[\begin{array}{l} u_{1}^{2}\\ u_{2}^{2}\\ \vdots \\ u_{m}^{2} \end{array}\right]+a_{3}\left[\begin{array}{l} u_{1}+\sum_{i=1}^{n}\rho_{i}P_{i}\left[u\right]\\ u_{2}+\sum_{i=1}^{n}\rho_{i}P_{i}\left[u\right]\\ \vdots \\ u_{m}+\sum_{i=1}^{n}\rho_{i}P_{i}\left[u\right] \end{array}\right]$$

### 2.2 Parameter identification

Because the MPI model is non-linear and non-differentiable, the parameter identification method of traditional linear model can't be applied directly. In this paper, particle swarm optimization (PSO) is used, which is a popular optimization algorithm. The objective function f(X) is expressed as follows:

(6) $$f\left(X\right)=\frac{1}{N}\sum_{i=1}^{N}\left(y_{i}-{\tilde{y}}_{i}\right)$$

where *N* is the size of the data, $X=[a_{1},a_{2},a_{3},a,a^{\prime },b,$ $\ldots b^{\prime },c,c^{\prime },\rho_{1}\cdots \rho_{n}]$is parameters to be identified, $y_{i}$and ${\tilde{y}}_{i}$ are the output predicted by the MPI model and the actual system, respectively. The basic PSO formulas can be expressed by the velocity and position as follows:

(7) $$\left\{ \begin{array}{l} V_{i}^{t+1}=\omega V_{i}^{t}+c_{1}r_{1}^{t}\left(P_{besti}^{t}-X_{i}^{t}\right)+c_{2}r_{2}^{t}\left(G_{best}^{t}-X_{i}^{t}\right)\\ X_{i}^{t+1}=X_{i}^{t}+V_{i}^{t+1} \end{array}\right.$$

where $r_{i}=rand_{i}\left(\right)$, i=1,2,⋯, are random numbers between 0 and 1. ω is the inertia weight, $c_{1}$and$c_{2}$ are local search acceleration coefficient and global search ace-leration coefficient, respectively. If the particle is in the historical optimal position for a long time, the second and third terms in Eq. (7) tend to 0, which means$X_{i}^{t}\to P_{besti}^{t}$. This will lead to a local optimization, rather than the global optimization. In order to avoid this situation, an improved rate update method is proposed in this paper. The swarm not only follows the historical best position and global best position, but also adds the information of the optimal position of a particle which is selected randomly from the group. In addition, since the inertia weight affects the search area, the large inertia value and the small inertia value are conducive to global search and local search, so ω is selected as the inertial weight of dynamic nonlinear attenuation and it can be expressed by:

(8) $$\omega_{i}=\omega_{\min}\;\exp\left(\frac{\omega_{\max}-\omega_{\min}}{T}\left(T-t_{i}\right)\right),i=1,2,\cdots ,T$$

where $\omega_{\max}$ and $\omega_{\min}$ are the maximum and minimum inertia weights respectively; $t_{i}$represents the time of the *i*th particle and T is the maximum number of iterations. Based on the above design, the modify PSO (MPSO) with velocity update formula is expressed as follows:

(9) $$V_{i}^{t+1}=\omega_{\min}\;\exp\left(\frac{\omega_{\max}-\omega_{\min}}{T}\left(T-t_{i}\right)\right)V_{i}^{t}+c_{1}r_{1}^{t}\left(P_{besti}^{t}-X_{i}^{t}\right)+c_{2}r_{2}^{t}\left(G_{best}^{t}-X_{i}^{t}\right)+c_{3}r_{3}^{t}\left(Q_{besti}^{t}-X_{i}^{t}\right)$$

where $c_{3}$ is the acceleration coefficient, $r_{3}=rand\left(\right)$ is a random number between 0 and 1; $Q_{besti}^{t}$is the optimal position information of a particle which is selected randomly.

Step 1: Set the number of operators.
Step 2: Initialize the parameters of particle swarm such as particle swarm size, maximum number of iterations, various acceleration coefficients, maximum and minimum weight values, velocity and position boundary values, etc.
Step 3: Initialize the velocity and position of each particle with the following formula

(10) $$\left\{ \begin{array}{l} X_{i}=X_{\min}+rand\left(\right)\left(X_{\max}-X_{\min}\right)\\ V_{i}=V_{\min}+rand\left(\right)\left(V_{\max}-V_{\min}\right) \end{array}\right.$$

Step 4: Calculate the fitness of each particle according to (7).
Step 5: Search for individual optimal position and global optimal position.

(11) $$pbest_{i}(t+1)=\left\{ \begin{array}{ll} pbest_{i}(t) & f(X_{i}(t+1)\geq f(\mathrm{pbes}t_{i})\\ X_{i}(t+1) & f(X_{i}(t+1)<f(\mathrm{pbes}t_{i}) \end{array}\right.$$

(12) $$gbest\left(k+1\right)=\min\left(pbest_{i}\left(k+1\right)\right)$$

Step 6: Update the velocity and location of each particle.
Step 7: Check whether the termination condition is satisfied. If satisfied the optimization is terminated and if not go to step 4.

In order to improve the searching efficiency of the MPSO, the parameters in the envelope function are limited in a range in advance according to the experimental data and experience.

### 2.3 Hysteresis inverse compensation based on the MPI model

The inverse model is used as the feedforward controller to linearize the input and output. A hysteresis compensation model based on MPI model is proposed. The inverse model is also PI type, which can be as:

(13) $$u\left(t\right)=h^{\prime }\left(y_{d}\right)+\sum_{i=1}^{n}\rho_{i}^{\prime }{P^{\prime }}^{-1}\left[y_{d}\right]\left(t\right)$$

where$h^{\prime }\left(y_{d}\right)=a_{1}^{\prime }y_{d}^{3}+a_{2}^{\prime }y_{d}^{2}+a_{3}^{\prime }y_{d}$, the$a_{1}^{\prime },a_{2}^{\prime },a_{3}^{\prime }$ are polynomial coefficients which need to be determined. The analytical inverse of the MPI model can be described as follows:

(14) $$\begin{array}{l} \rho_{0}^{\prime }=\frac{1}{\rho_{0}}\\ y_{d0}^{i}=\sum_{j=0}^{i}\rho_{j}y_{0}^{j}+\sum_{j=i+1}^{n}\rho_{j}y_{0}^{j}\\ \rho_{i}^{\prime }=\frac{-\rho_{i}}{\left(\sum_{j=0}^{i}\rho_{j}\right)\left(\sum_{j=0}^{i-1}\rho_{j}\right)}i=1,\ldots ,n \end{array}$$

## 3 Controller design

**Figure 1:**
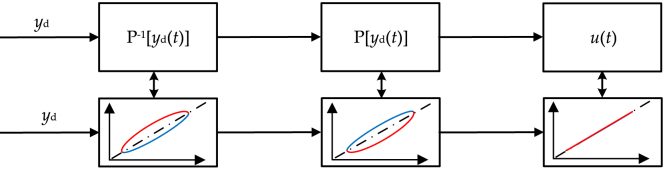
The schematic of feedforward control.

In order to improve the dynamic positioning accuracy of the piezoelectric actuator, reduce the parameter uncertainty, compensation error and external interference of the piezoelectric actuator, the driving system of the piezo-electric actuator is modeled, and an adaptive backstep sliding mode controller is designed. The stability of the control system is proved by choosing appropriate Lyapunov function and intermediate dummy variable (see Figure 1).

### 3.1 Model of piezoelectric actuator

The driving system of piezoelectric actuator is mainly composed of mechanical and electrical parts. Due to the complex structure of piezoelectric drive system, this paper establishes the hysteresis model of piezoelectric drive from mechanical and electrical aspects, which is convenient for subsequent modeling and analysis.

This chapter introduces the drive circuit and control circuit of piezoelectric driver in detail. The dynamic equation from the input stage to the output stage can be expressed by the following three transfer functions:

(15) $$\begin{array}{l} G_{1}\left(s\right)=\frac{u_{K}\left(s\right)}{u_{in}\left(s\right)}=K_{1}\\ G_{2}\left(s\right)=\frac{T_{e}}{RCs+1}\\ G_{3}\left(s\right)=K_{S} \end{array}$$

where $u_{in}$ is the input; $u_{K}$is the voltage due to this effect; $u_{S}$is the voltage; P[u]is the hysteresis effect; CandRare the total capacitance and resistance connected in parallel with the electromechanical transformer, which have a ratio of $T_{e}$; $K_{V}$and $K_{S}$represent voltage and power amplification of the driving circuit. According to Newton's law, the mechanical model can be written as follows:

(16) $$\begin{array}{l} F_{t}=T_{e}u\\ m\ddot{x}+b\dot{x}+kx=F_{t}+F_{e} \end{array}$$

where $F_{e}$and$F_{t}$ are the external force and the driving force, respectively; mis the mass of the piezoelectric actuator. Due to the intrinsic hysteretic nonlinearity and tracking performance of piezoelectric actuator is mainly focused in this paper, the external force on the piezoelectric actuator is temporarily ignored that is $F_{e}=0$.

(17) $$m\ddot{x}+b\dot{x}+kx=T_{e}u$$

After Laplace transformation, the transfer function can be expressed as

(18) $$G_{4}\left(s\right)=\frac{T_{e}}{ms^{2}+bs+k}$$

Combining Eq. (18) with Eq. (21), we can obtain

(19) $$G\left(s\right)=G_{1}\left(s\right)G_{2}\left(s\right)G_{3}\left(s\right)G_{4}\left(s\right)=K_{1}\frac{T_{e}}{RCs+1}K_{S}\frac{T_{e}}{ms^{2}+bs+k}=\frac{K_{S}K_{1}T_{e}^{2}}{\left(RCs+1\right)\left(\rho h\pi r^{2}s^{2}+bs+k\right)}$$

In this experiment, the parameters are listed in Table 1.

**Table 1:** Model parameters.

Number	r_i_	b_i_	a_i_
1	0.1	0.2054	0.2547
2	0.2	0.1985	-0.00358
3	0.3	0.0237	0.7123
4	0.4	0.0148	
5	0.5	0.0456	
6	0.6	0.0645	
7	0.7	0.0051	
8	0.8	0.0014	
9	0.9	0.0033	

Name	Value
K_1_	20
K_S_	7.6
ρ	7700
h	0.005
r	0.0064
Te	4.6
C	0.0000072
R	10
b	61
k	428155

### 3.2 Modeling uncertainties

The system model is as follows:

(20) $$\left\{ \begin{array}{l} x_{1}=x\\ x_{2}=\dot{x}\\ x_{3}=\ddot{x} \end{array}\right.$$

according to equation (19), equation (20) can be written as follows:

(21) $$\left\{ \begin{array}{l} {\dot{x}}_{1}=x_{2}\\ {\dot{x}}_{2}=x_{3}\\ {\dot{x}}_{3}=a_{1}x_{1}+a_{2}x_{2}+a_{3}x_{3}+u+d_{n}+d \end{array}\right.$$

where$x_{1}=y$, $d_{n}$is the feedforward compensation error, dis external disturbance. Both of them are bounded disturbances.

(22) $$\left\{ \begin{array}{l} \left|d_{n}\right|\leq \tilde{N}\\ \left|d\right|\leq \tilde{D} \end{array}\right.$$

where $\tilde{N}$ and $\tilde{D}$ are the infinities of nonlinear error and interference.

### 3.3 Sliding mode controller

The coefficients $a_{1},a_{2},a_{3}$ can be obtained as follows:

(23) $$\left\{ \begin{array}{l} a_{1}=RC\rho h\pi r^{2}\\ a_{2}=RCb+\rho h\pi r^{2}\\ a_{3}=RCk+b \end{array}\right.$$

The system tracking error is defined by

(24) $$\left\{ \begin{array}{l} z_{1}=x_{1}-x_{d}\\ z_{2}=x_{2}-\alpha_{1}\\ z_{3}=x_{3}-\alpha_{2} \end{array}\right.$$

where $x_{d}$is the expected value, $\alpha_{1}$ and $\alpha_{2}$ are the control laws to be selected, and the derivative of $z_{1}$ can be written as follows:

(25) $${\dot{z}}_{1}={\dot{x}}_{1}-{\dot{x}}_{d}=x_{2}-{\dot{x}}_{d}=z_{2}+\alpha_{1}-{\dot{x}}_{d}$$

Choosing the Lyapunov function:

(26) $$V_{1}=\frac{1}{2}z_{1}^{2}$$

the derivative of Eq. (26) can be obtained as follows:

(27) $${\dot{V}}_{1}=z_{1}{\dot{z}}_{1}=z_{1}\left(z_{2}+\alpha_{1}-{\dot{x}}_{d}\right)$$

Choosing $\alpha_{1}=-c_{1}z_{1}+{\dot{x}}_{d}$, $c_{1}>0$.

(28) $${\dot{V}}_{1}=z_{1}{\dot{z}}_{1}=z_{1}\left(z_{2}-c_{1}z_{1}+{\dot{x}}_{d}-{\dot{x}}_{d}\right)=-c_{1}z_{1}^{2}+z_{1}z_{2}$$

the derivative of $z_{2}$ can be expressed:

(29) $${\dot{z}}_{2}={\dot{x}}_{2}-{\dot{\alpha }}_{1}=x_{3}-{\dot{\alpha }}_{1}$$

choosing the Lyapunov function:

(30) $$V_{2}=\frac{1}{2}z_{2}^{2}$$

the derivative of Eq. (30) can be obtained as follows:

(31) $${\dot{V}}_{2}=z_{2}{\dot{z}}_{2}=z_{2}\left(x_{3}-{\dot{\alpha }}_{1}\right)=z_{2}\left(z_{3}+\alpha_{2}-{\dot{\alpha }}_{1}\right)$$

Let $\alpha_{2}=-c_{2}z_{2}-z_{1}+{\dot{\alpha }}_{1}$, $c_{2}>0$ Eq. (31) can be rewritten as:

(32) $${\dot{V}}_{2}=z_{2}\left(z_{3}+\alpha_{2}-{\dot{\alpha }}_{1}\right)=-c_{2}z_{2}^{2}-z_{1}z_{2}+z_{2}z_{3}$$

the derivative of $z_{3}$ can be expressed as follows:

(33) $${\dot{z}}_{3}={\dot{x}}_{3}-{\dot{\alpha }}_{2}=a_{1}x_{1}+a_{2}x_{2}+a_{3}x_{3}+u+d_{n}+d-{\dot{\alpha }}_{2}$$

Define the sliding surface of the system:

(34) $$s=k_{1}{\dot{z}}_{1}+k_{2}z_{1}+z_{3}$$

where $k_{1}>0$and$k_{2}>0$ are constants. The condition that the system reaches the sliding surface is$s\dot{s}<0$, choose the Lyapunov function:

(35) $$V_{3}=\frac{1}{2}z_{1}^{2}+\frac{1}{2}z_{2}^{2}+\frac{1}{2}s^{2}$$

The derivative of Eq. (35) can be obtained

(36) $$\begin{array}{cc} {\dot{V}}_{3} & =-c_{1}z_{1}^{2}-c_{2}z_{2}^{2}-k_{1}z_{2}^{2}+s\left(k_{1}x_{3}-k_{1}{\dot{\alpha }}_{1}+{\dot{x}}_{3}-{\dot{\alpha }}_{2}+x_{2}-\alpha_{1}\right)\\ & =-c_{1}z_{1}^{2}-c_{2}z_{2}^{2}-k_{1}z_{2}^{2}+s\left(k_{1}x_{3}-k_{1}{\dot{\alpha }}_{1}+a_{1}x_{1}+a_{2}x_{2}+\cdots a_{3}x_{3}+u+d_{n}+d-{\dot{\alpha }}_{2}+x_{2}-\alpha_{1}\right) \end{array}$$

### 3.4 Proof of stability

Define$a_{1},a_{2},a_{3}$as the estimation errors

(37) $$\left\{ \begin{array}{l} {\tilde{a}}_{1}=a_{1}-{\hat{a}}_{1}\\ {\tilde{a}}_{2}=a_{2}-{\hat{a}}_{2}\\ {\tilde{a}}_{3}=a_{3}-{\hat{a}}_{3} \end{array}\right.$$

where ${\hat{a}}_{1},{\hat{a}}_{2},{\hat{a}}_{3}$are estimates of the coefficients respe-ctively ${\tilde{a}}_{1},{\tilde{a}}_{2},{\tilde{a}}_{3}$are estimation errors. All coefficients are bounded estimates. Choose the Lyapunov function:

(38) $$V=V_{3}+\frac{1}{2a_{1}\lambda }{\tilde{a}}_{1}^{2}+\frac{1}{2a_{2}\beta }{\tilde{a}}_{2}^{2}+\frac{1}{2a_{3}\eta }{\tilde{a}}_{3}^{2}$$

the derivative of Eq. (38) can be expressed as:

(39) $$\dot{V}=-c_{1}z_{1}^{2}-(c_{2}+k_{1})z_{2}^{2}+s(a_{1}x_{1}+(a_{2}+1)x_{2}+(a_{3}+k_{1})x_{3}+\cdots u+d_{n}+d-{\dot{\alpha }}_{2}-k_{1}{\dot{\alpha }}_{1}-\alpha_{1})+\frac{1}{a_{1}\lambda }{\tilde{a}}_{1}\left(-{\dot{\hat{a}}}_{1}\right)+\frac{1}{a_{2}\beta }{\tilde{a}}_{2}\left(-{\dot{\hat{a}}}_{2}\right)+\frac{1}{a_{3}\eta }{\tilde{a}}_{3}\left(-{\dot{\hat{a}}}_{3}\right)$$

define$\left\{ \begin{array}{l} u=-{\hat{a}}_{1}x_{1}-\left({\hat{a}}_{2}+1\right)x_{2}-\left({\hat{a}}_{3}+k_{1}\right)x_{3}-\ldots \\ k_{3}s-\tilde{N}\mathrm{sgn}\left(s\right)-\tilde{D}\mathrm{sgn}\left(s\right)+ϒ\\ ϒ={\dot{\alpha }}_{2}+k_{1}{\dot{\alpha }}_{1}+\alpha_{1} \end{array}\right.$then we have

(40) $$\dot{V}=-c_{1}z_{1}^{2}-\left(c_{2}+k_{1}\right)z_{2}^{2}+s[\left(a_{1}-{\hat{a}}_{1}\right)x_{1}+\left(a_{2}-{\hat{a}}_{2}\right)x_{2}+\left(a_{3}-{\hat{a}}_{3}\right)x_{3}-k_{3}s-\tilde{N}\mathrm{sgn}\left(s\right)-\tilde{D}\mathrm{sgn}\left(s\right)+d_{n}+d]+\frac{1}{a_{1}\lambda }{\tilde{a}}_{1}\left(-{\dot{\hat{a}}}_{1}\right)+\frac{1}{a_{2}\beta }{\tilde{a}}_{2}\left(-{\dot{\hat{a}}}_{2}\right)+\frac{1}{a_{3}\eta }{\tilde{a}}_{3}\left(-{\dot{\hat{a}}}_{3}\right)$$

selecting the adaptive control law, $\left\{ \begin{array}{l} {\dot{\hat{a}}}_{1}=sx_{1}a_{1}\lambda \\ {\dot{\hat{a}}}_{2}=sx_{2}a_{2}\beta \\ {\dot{\hat{a}}}_{3}=sx_{3}a_{3}\eta \end{array}\right.$, since

$\left\{ \begin{array}{l} \tilde{N}\left|s\right|\geq d_{n}s\\ \tilde{D}\left|s\right|\geq ds \end{array}\right.$, Eq. (40) can be rewritten as follows:

(41) $$\dot{V}=-c_{1}z_{1}^{2}-\left(c_{2}+k_{1}\right)z_{2}^{2}-k_{3}s^{2}+{\tilde{a}}_{1}\left(sx_{1}-\frac{{\dot{\hat{a}}}_{1}}{a_{1}\lambda }\right)+\ldots {\tilde{a}}_{2}\left(sx_{2}-\frac{{\dot{\hat{a}}}_{2}}{a_{2}\beta }\right)+{\tilde{a}}_{3}\left(sx_{3}-\frac{{\dot{\hat{a}}}_{3}}{a_{3}\eta }\right)-\tilde{N}\left|s\right|-\tilde{D}\left|s\right|+d_{n}s+ds\leq -c_{1}z_{1}^{2}-\left(c_{2}+k_{1}\right)z_{2}^{2}-k_{3}s^{2}\leq 0$$

The system is asymptotically stable. It means that the system can track the desired signal asymptotically.

## 4 Experiment

### 4.1 Experiment setup

**Figure 2:**
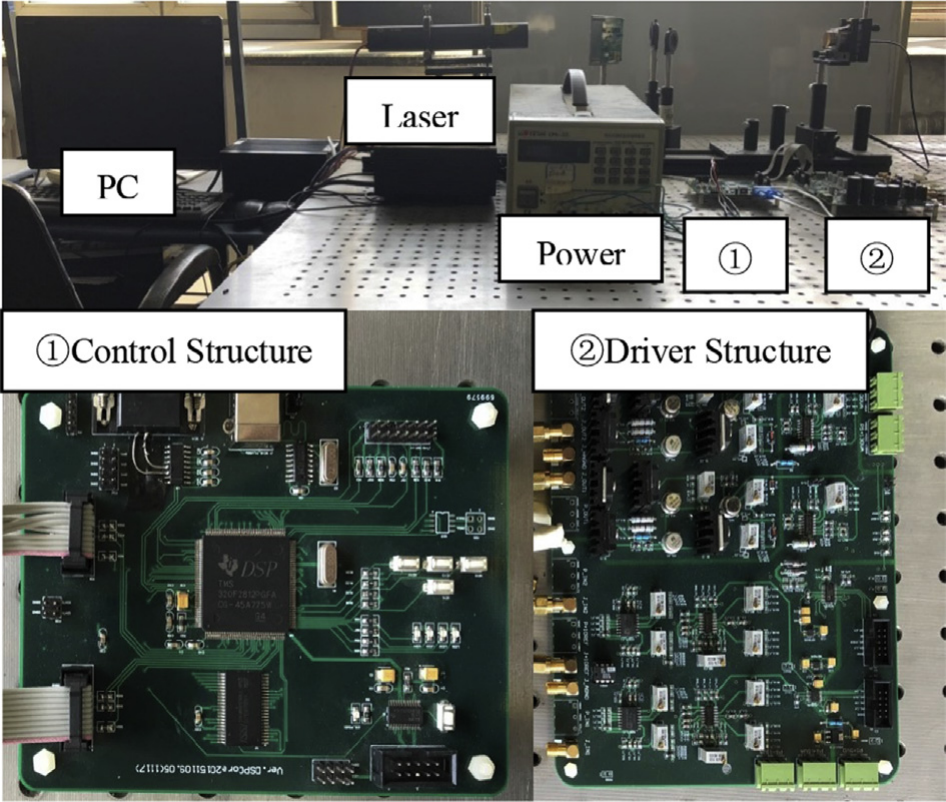
Piezoelectric driving system.

Piezoelectric ceramic driver is S-330.2SL piezoelectric ceramic driver produced by Physik instrument company. It has the characteristics of maximum angle, equivalent capacity of each axis 3uF and fast response time. The power supply voltage is 100V, the control voltage is 0–100V, and the feedback structure is resistance strain sensor. The voltage corresponding to the deformation position of the current piezoelectric driver is feedback to the circuit. Figure 2 shows the piezoelectric driver control system, mainly divided into control circuit and drive circuit. The control circuit processes the input signal and the drive circuit amplifies the control signal to drive the piezoelectric driver.

### 4.2 Experiment results

**Figure 3:**
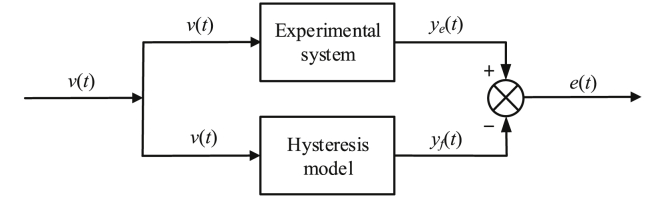
Error of the fitting model.

Firstly, the error of the fitting model is tested in the experiment as shown in Figure 3, where v(t)is expected control input, $y_{e}\left(t\right)$is the experimental output, $y_{f}\left(t\right)$is output of the model, and e(t)is error.

**Figure 4:**
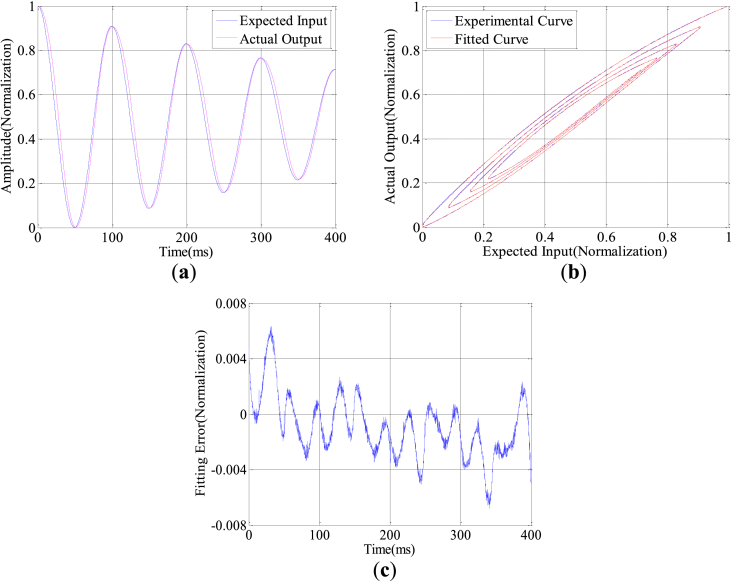
Model validation on sinusoidal control signals of 10 Hz with variable amplitude(Norma-lization): (a) Expected input and actual output curves with variable amplitude; (b) Experimental and fitting curves with variable amplitude; (c) Model fitting error with variable amplitude.

The constant amplitude and variable amplitude sinusoidal signals of 10Hz are applied to the system, and the proposed model fitting method is verified. The results are shown in Figure 4. The hysteresis codes used in this work can be seen in the supplementary file code.rar.

In this paper, in addition to the experiment of 10 Hz sinusoidal control signal, other frequency control signals are tested and analyzed. The maximum error and mean square error of the fitting model at other frequencies are shown in Table 2.

**Table 2:** Model fitting maximum error and mean square error.

Control signals (Hz)	Max error (%)	Mean square error (%)
2Hz constant amplitude	0.31	0.21
5Hz constant amplitude	0.43	0.25
10Hz constant amplitude	0.49	0.18
20Hz constant amplitude	0.69	0.35
50Hz constant amplitude	1.29	0.36
100Hz constant amplitude	0.92	0.36
5Hz variable amplitude	0.47	0.21
10Hz variable amplitude	0.64	0.22
100Hz variable amplitude	1.64	0.62

**Figure 5:**
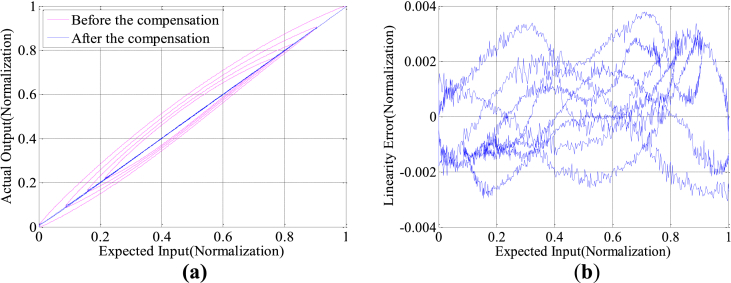
Feedforward compensation experiment with 10 Hz variable amplitude sine input: (a) Feedforward compensation before and after hysteresis curve with variable amplitude; (b) Linearity error with variable amplitude.

Secondly, the constant amplitude and variable amplitude sinusoidal signals of 10Hz are applied to the system to verify the effectiveness of the feedforward linearization compensation method (see Figure 5).

**Figure 6:**
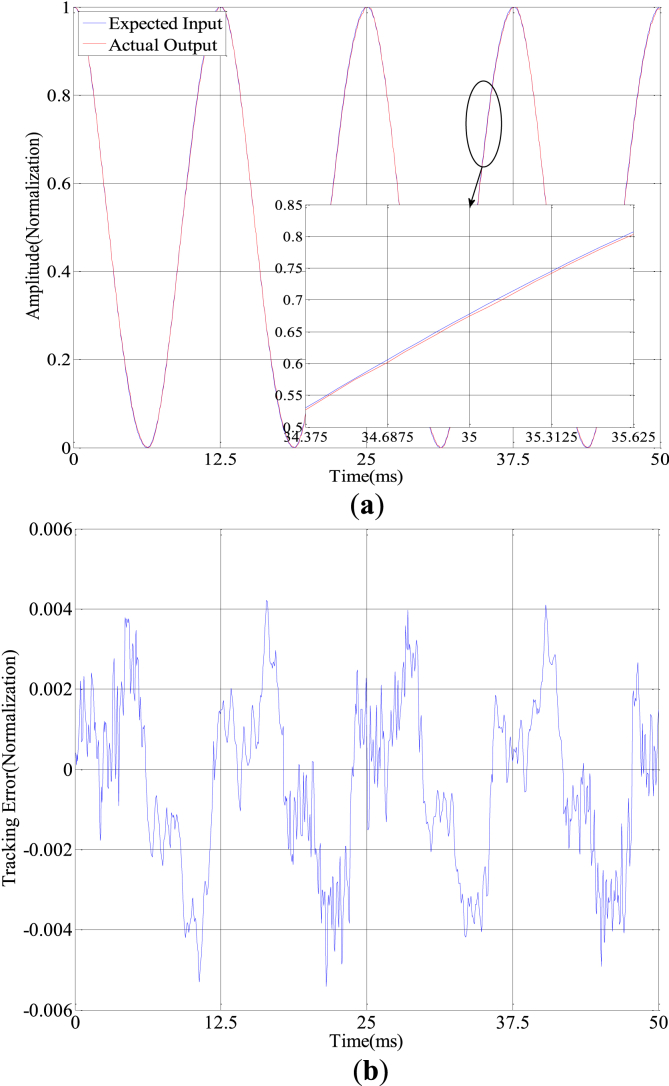
Precision tracking experiment with 80Hz constant amplitude sine input: (a) Curve of expect and tracking with constant amplitude; (b) Tracking error with constant amplitude.

**Figure 7:**
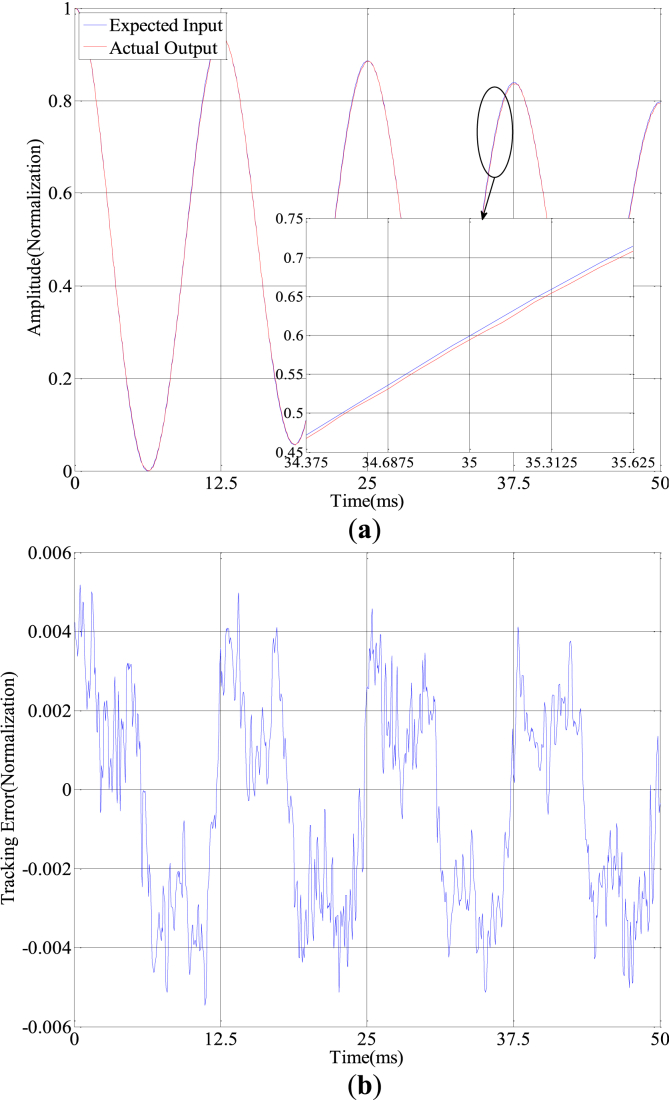
Precision tracking experiment with 80 Hz variable amplitude sine input: (a) Curve of expect and tracking with variable amplitude; (b) Tracking error with variable amplitude.

The constant amplitude and variable amplitude sinusoids with 80Hz are applied on the system for evaluating the effectiveness of the proposed control strategy (see Figures 6 and 7 and Tables 3 and 4).

**Table 3:** Model fitting linearity standard error.

Control signals (Hz)	Error without compensation (%)	Error with compensation (%)
10 Hz constant amplitude	5.53	0.17
20 Hz constant amplitude	7.44	0.38
50 Hz constant amplitude	8.56	0.53
100 Hz constant amplitude	13.05	0.67
10 Hz variable amplitude	7.37	0.36
100 Hz variable amplitude	14.88	0.74

**Table 4:** Error analysis table.

Constant amplitude sine input		
Control method	Max error (Normalization)	Mean square error
No compensation	0.0784	0.0348
Feedforward compensation	0.0099	0.0035
Feedforward and feedback compensation	0.0054	0.0021

Variable amplitude sine input		
No compensation	0.1199	0.0428
Feedforward compensation	0.0118	0.0040
Feedforward and feedback compensation	0.0055	0.0026

## 5 Conclusion

In this paper, a MPI model with generalized clearance operator is proposed, and a feedforward compensation controller is designed to reduce the asymmetric hysteresis effect. In order to reduce the compensation error, an adaptive backstepping sliding mode controller is designed. By choosing appropriate Lyapunov function and intermediate dummy variable, the stability of the system is proved. The experimental results show that the linearity error of the system is reduced from 10% to less than 1%, and the tracking error is reduced by 90%.

## Declarations

### Author contribution statement

Zhiliang Yu: Conceived and designed the experiments; Performed the experiments; Analyzed and interpreted the data.

Yue Wu: Analyzed and interpreted the data; Wrote the paper.

Zhiyi Fang & Hailin Sun: Contributed reagents, materials, analysis tools or data.

### Funding statement

This work was supported by the 10.13039/100014717National Natural Science Foundation of China under grants No. 61503096 and the 10.13039/501100004027Hei Long Jiang Postdoctoral Foundation under Grant No. LBHZ14101.

### Competing interest statement

The authors declare no conflict of interest.

### Additional information

No additional information is available for this paper.

## Appendix A Supplementary data

Code.rar
